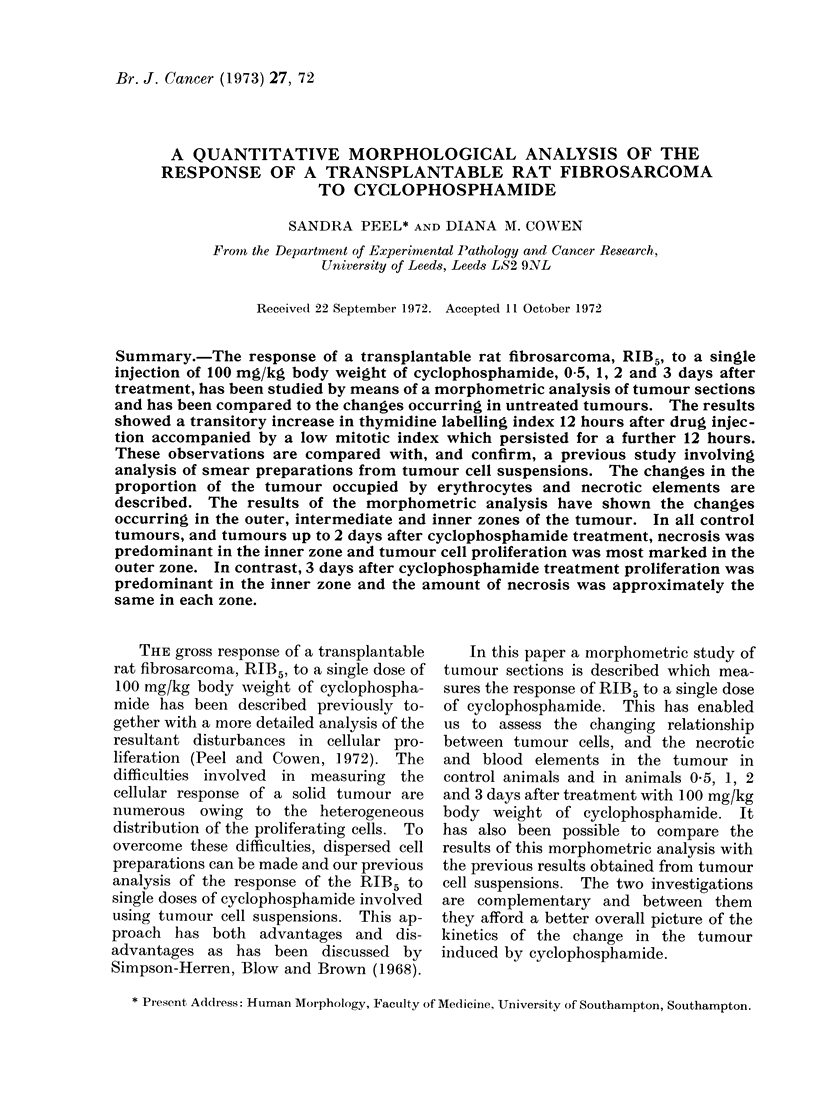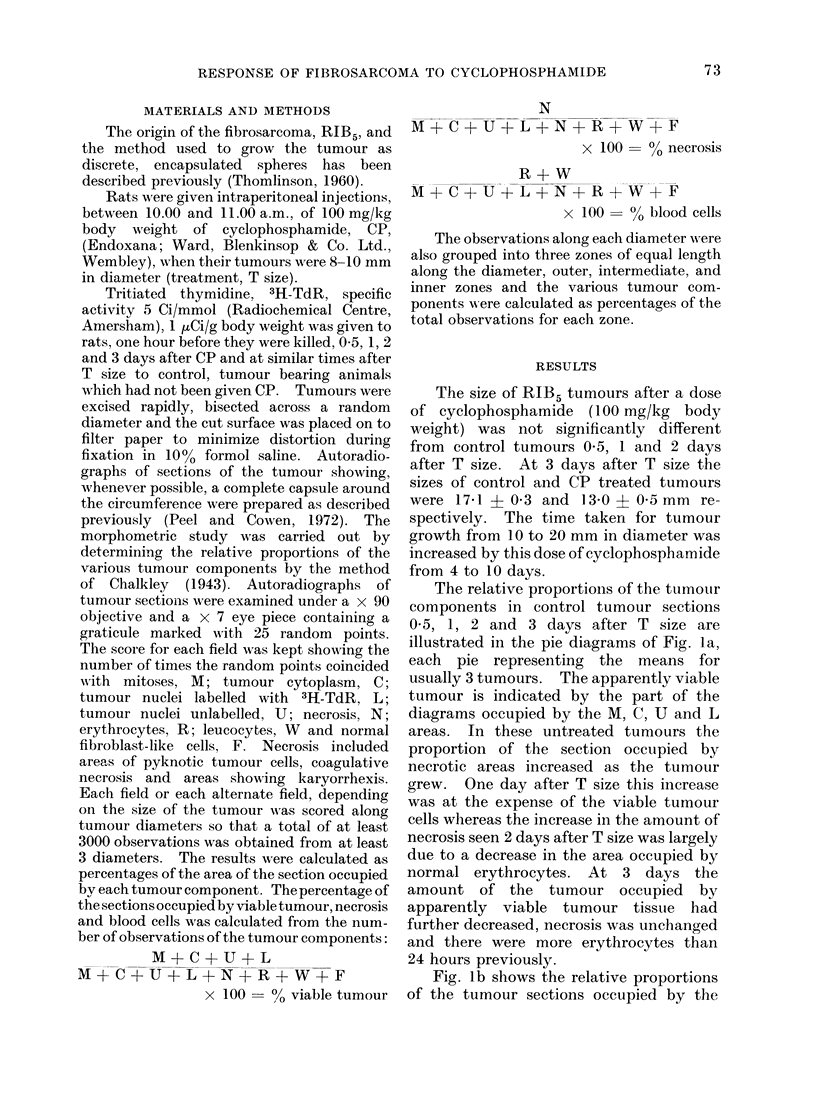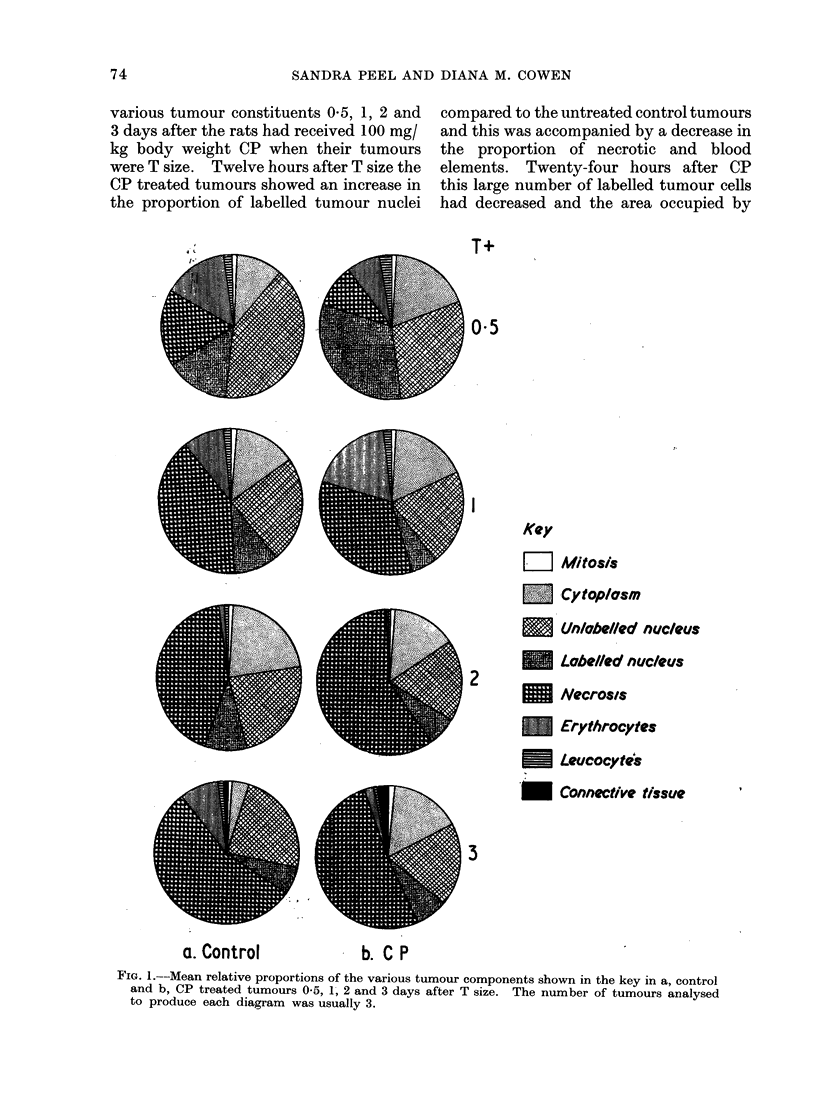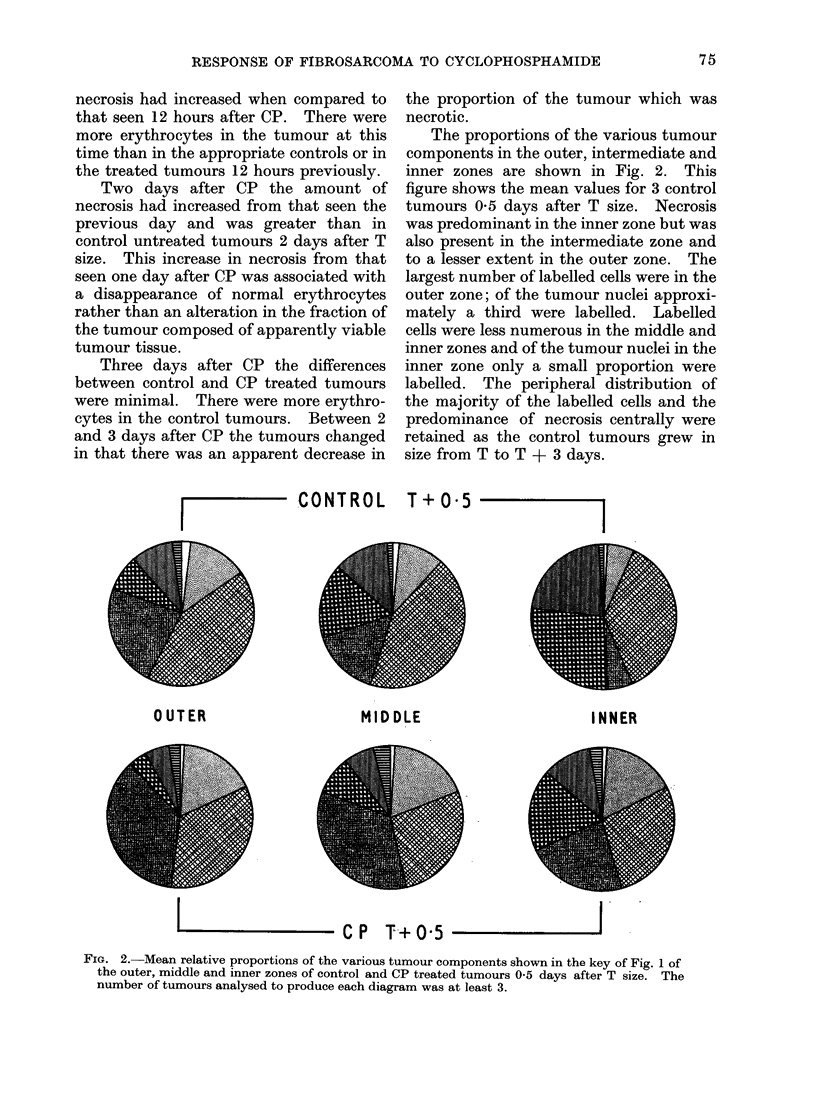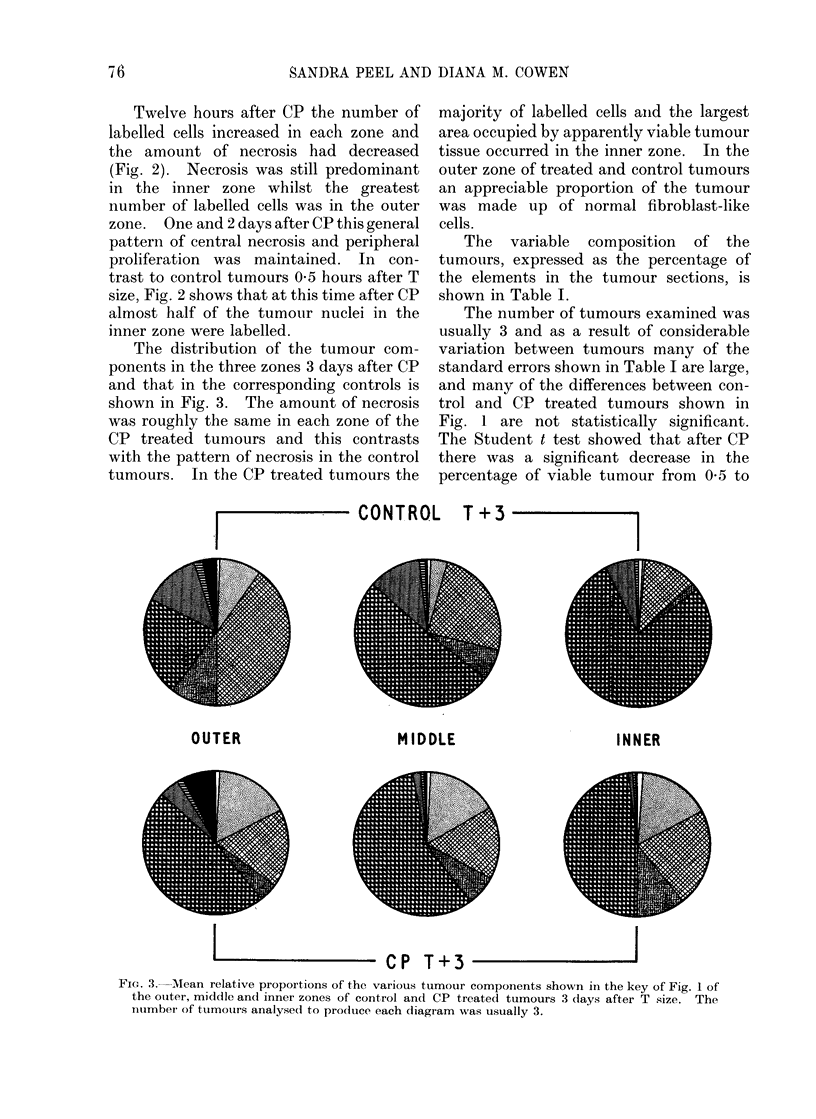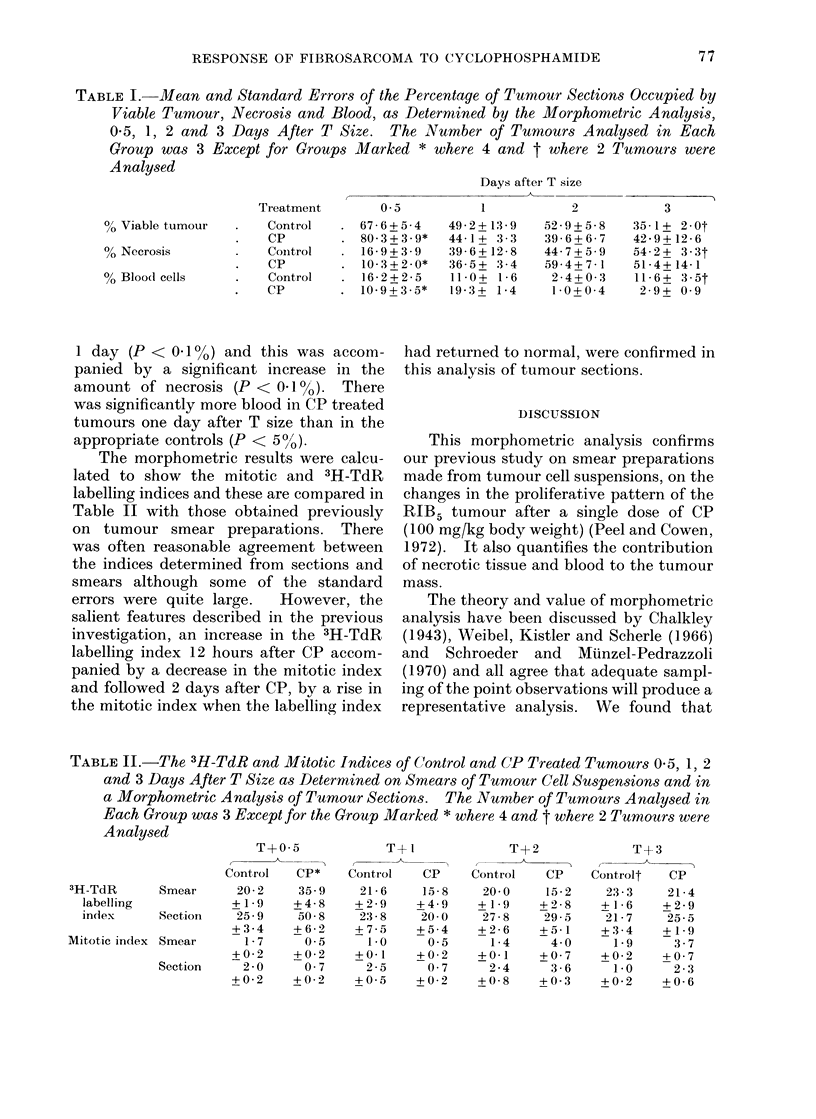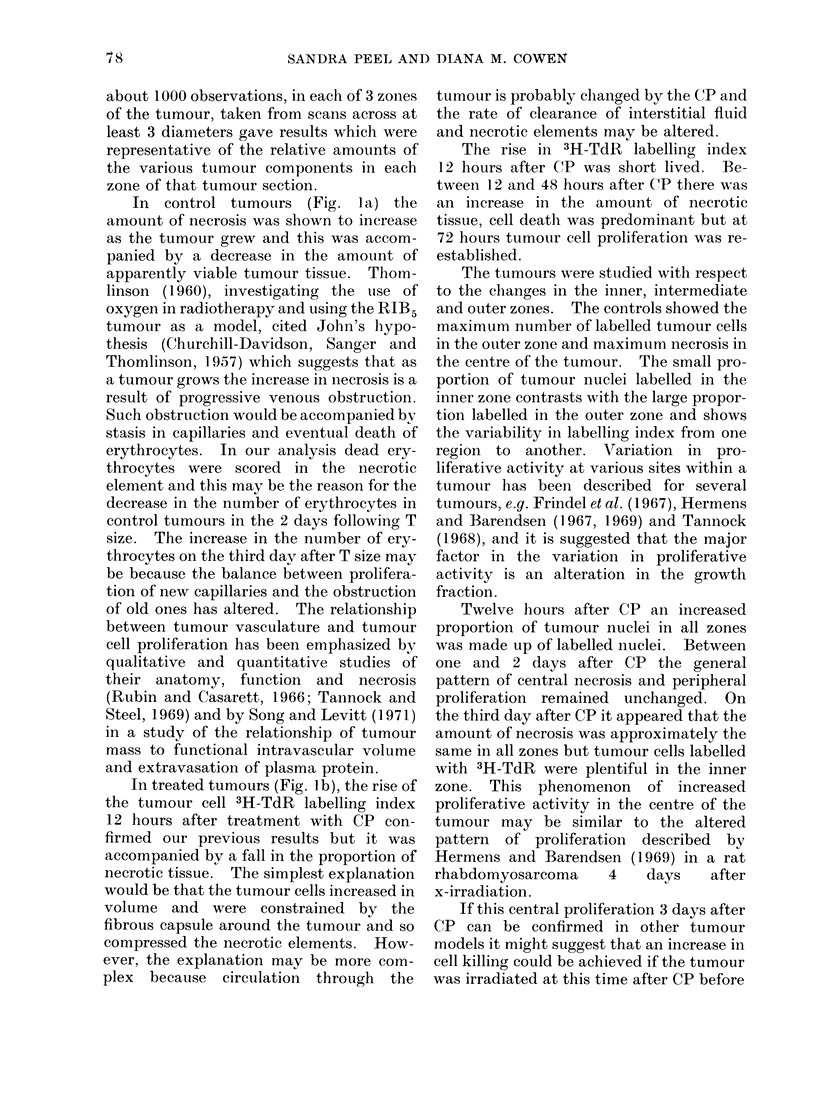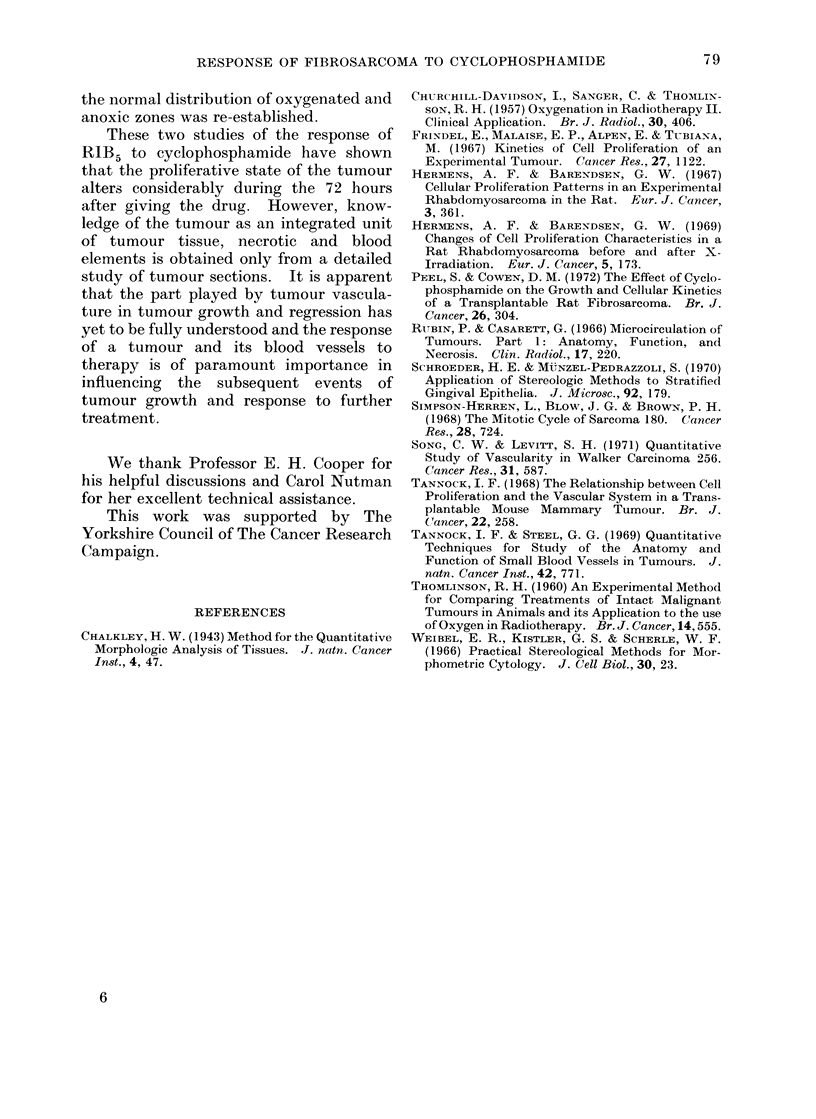# A Quantitative Morphological Analysis of the Response of a Transplantable Rat Fibrosarcoma to Cyclophosphamide

**DOI:** 10.1038/bjc.1973.9

**Published:** 1973-01

**Authors:** Sandra Peel, Diana M. Cowen

## Abstract

The response of a transplantable rat fibrosarcoma, RIB_5_, to a single injection of 100 mg/kg body weight of cyclophosphamide, 0·5, 1, 2 and 3 days after treatment, has been studied by means of a morphometric analysis of tumour sections and has been compared to the changes occurring in untreated tumours. The results showed a transitory increase in thymidine labelling index 12 hours after drug injection accompanied by a low mitotic index which persisted for a further 12 hours. These observations are compared with, and confirm, a previous study involving analysis of smear preparations from tumour cell suspensions. The changes in the proportion of the tumour occupied by erythrocytes and necrotic elements are described. The results of the morphometric analysis have shown the changes occurring in the outer, intermediate and inner zones of the tumour. In all control tumours, and tumours up to 2 days after cyclophosphamide treatment, necrosis was predominant in the inner zone and tumour cell proliferation was most marked in the outer zone. In contrast, 3 days after cyclophosphamide treatment proliferation was predominant in the inner zone and the amount of necrosis was approximately the same in each zone.


Br. J. Cancer (1973) 27, 72

### A QUANTITATIVE MORPHOLOGICAL ANALYSIS OF THE RESPONSE OF A TRANSPLANTABLE RAT FIBROSARCOMA TO CYCLOPHOSPHAMIDE

SANDRA PEEL* AND DIANA M. COWEN

*From the Department of Experimental Pathology and Cancer Research, University of Leeds, Leeds LS2 9NL*

Received 22 September 1972. Accepted 11 October 1972

**Summary.—The response of a transplantable rat fibrosarcoma, $RIB_5$, to a single injection of 100 mg/kg body weight of cyclophosphamide, 0·5, 1, 2 and 3 days after treatment, has been studied by means of a morphometric analysis of tumour sections and has been compared to the changes occurring in untreated tumours. The results showed a transitory increase in thymidine labelling index 12 hours after drug injection accompanied by a low mitotic index which persisted for a further 12 hours. These observations are compared with, and confirm, a previous study involving analysis of smear preparations from tumour cell suspensions. The changes in the proportion of the tumour occupied by erythrocytes and necrotic elements are described. The results of the morphometric analysis have shown the changes occurring in the outer, intermediate and inner zones of the tumour. In all control tumours, and tumours up to 2 days after cyclophosphamide treatment, necrosis was predominant in the inner zone and tumour cell proliferation was most marked in the outer zone. In contrast, 3 days after cyclophosphamide treatment proliferation was predominant in the inner zone and the amount of necrosis was approximately the same in each zone.**

THE gross response of a transplantable rat fibrosarcoma, $RIB_5$, to a single dose of 100 mg/kg body weight of cyclophosphamide has been described previously together with a more detailed analysis of the resultant disturbances in cellular proliferation (Peel and Cowen, 1972). The difficulties involved in measuring the cellular response of a solid tumour are numerous owing to the heterogeneous distribution of the proliferating cells. To overcome these difficulties, dispersed cell preparations can be made and our previous analysis of the response of the $RIB_5$ to single doses of cyclophosphamide involved using tumour cell suspensions. This approach has both advantages and disadvantages as has been discussed by Simpson-Herren, Blow and Brown (1968).

In this paper a morphometric study of tumour sections is described which measures the response of $RIB_5$ to a single dose of cyclophosphamide. This has enabled us to assess the changing relationship between tumour cells, and the necrotic and blood elements in the tumour in control animals and in animals 0·5, 1, 2 and 3 days after treatment with 100 mg/kg body weight of cyclophosphamide. It has also been possible to compare the results of this morphometric analysis with the previous results obtained from tumour cell suspensions. The two investigations are complementary and between them they afford a better overall picture of the kinetics of the change in the tumour induced by cyclophosphamide.

* Present Address: Human Morphology, Faculty of Medicine, University of Southampton, Southampton.

### MATERIALS AND METHODS

The origin of the fibrosarcoma, $RIB_5$, and the method used to grow the tumour as discrete, encapsulated spheres has been described previously (Thomlinson, 1960).

Rats were given intraperitoneal injections, between 10.00 and 11.00 a.m., of 100 mg/kg body weight of cyclophosphamide, CP, (Endoxana; Ward, Blenkinsop & Co. Ltd., Wembley), when their tumours were 8–10 mm in diameter (treatment, T size).

Tritiated thymidine, $^3$H-TdR, specific activity 5 Ci/mmol (Radiochemical Centre, Amersham), 1 $\mu$Ci/g body weight was given to rats, one hour before they were killed, 0·5, 1, 2 and 3 days after CP and at similar times after T size to control, tumour bearing animals which had not been given CP. Tumours were excised rapidly, bisected across a random diameter and the cut surface was placed on to filter paper to minimize distortion during fixation in 10% formol saline. Autoradiographs of sections of the tumour showing, whenever possible, a complete capsule around the circumference were prepared as described previously (Peel and Cowen, 1972). The morphometric study was carried out by determining the relative proportions of the various tumour components by the method of Chalkley (1943). Autoradiographs of tumour sections were examined under a $\times$ 90 objective and a $\times$ 7 eye piece containing a graticule marked with 25 random points. The score for each field was kept showing the number of times the random points coincided with mitoses, M; tumour cytoplasm, C; tumour nuclei labelled with $^3$H-TdR, L; tumour nuclei unlabelled, U; necrosis, N; erythrocytes, R; leucocytes, W and normal fibroblast-like cells, F. Necrosis included areas of pyknotic tumour cells, coagulative necrosis and areas showing karyorrhexis. Each field or each alternate field, depending on the size of the tumour was scored along tumour diameters so that a total of at least 3000 observations was obtained from at least 3 diameters. The results were calculated as percentages of the area of the section occupied by each tumour component. The percentage of the sections occupied by viable tumour, necrosis and blood cells was calculated from the number of observations of the tumour components:

$$\frac{M + C + U + L}{M + C + U + L + N + R + W + F} \times 100 = \% \text{ viable tumour}$$

$$\frac{N}{M + C + U + L + N + R + W + F} \times 100 = \% \text{ necrosis}$$

$$\frac{R + W}{M + C + U + L + N + R + W + F} \times 100 = \% \text{ blood cells}$$

The observations along each diameter were also grouped into three zones of equal length along the diameter, outer, intermediate, and inner zones and the various tumour components were calculated as percentages of the total observations for each zone.

### RESULTS

The size of $RIB_5$ tumours after a dose of cyclophosphamide (100 mg/kg body weight) was not significantly different from control tumours 0·5, 1 and 2 days after T size. At 3 days after T size the sizes of control and CP treated tumours were 17·1 $\pm$ 0·3 and 13·0 $\pm$ 0·5 mm respectively. The time taken for tumour growth from 10 to 20 mm in diameter was increased by this dose of cyclophosphamide from 4 to 10 days.

The relative proportions of the tumour components in control tumour sections 0·5, 1, 2 and 3 days after T size are illustrated in the pie diagrams of Fig. 1a, each pie representing the means for usually 3 tumours. The apparently viable tumour is indicated by the part of the diagrams occupied by the M, C, U and L areas. In these untreated tumours the proportion of the section occupied by necrotic areas increased as the tumour grew. One day after T size this increase was at the expense of the viable tumour cells whereas the increase in the amount of necrosis seen 2 days after T size was largely due to a decrease in the area occupied by normal erythrocytes. At 3 days the amount of the tumour occupied by apparently viable tumour tissue had further decreased, necrosis was unchanged and there were more erythrocytes than 24 hours previously.

Fig. 1b shows the relative proportions of the tumour sections occupied by the various tumour constituents 0·5, 1, 2 and 3 days after the rats had received 100 mg/kg body weight CP when their tumours were T size. Twelve hours after T size the CP treated tumours showed an increase in the proportion of labelled tumour nuclei compared to the untreated control tumours and this was accompanied by a decrease in the proportion of necrotic and blood elements. Twenty-four hours after CP this large number of labelled tumour cells had decreased and the area occupied by

FIG. 1.—Mean relative proportions of the various tumour components shown in the key in a, control and b, CP treated tumours 0·5, 1, 2 and 3 days after T size. The number of tumours analysed to produce each diagram was usually 3.

necrosis had increased when compared to that seen 12 hours after CP. There were more erythrocytes in the tumour at this time than in the appropriate controls or in the treated tumours 12 hours previously.

Two days after CP the amount of necrosis had increased from that seen the previous day and was greater than in control untreated tumours 2 days after T size. This increase in necrosis from that seen one day after CP was associated with a disappearance of normal erythrocytes rather than an alteration in the fraction of the tumour composed of apparently viable tumour tissue.

Three days after CP the differences between control and CP treated tumours were minimal. There were more erythrocytes in the control tumours. Between 2 and 3 days after CP the tumours changed in that there was an apparent decrease in the proportion of the tumour which was necrotic.

The proportions of the various tumour components in the outer, intermediate and inner zones are shown in Fig. 2. This figure shows the mean values for 3 control tumours 0·5 days after T size. Necrosis was predominant in the inner zone but was also present in the intermediate zone and to a lesser extent in the outer zone. The largest number of labelled cells were in the outer zone; of the tumour nuclei approximately a third were labelled. Labelled cells were less numerous in the middle and inner zones and of the tumour nuclei in the inner zone only a small proportion were labelled. The peripheral distribution of the majority of the labelled cells and the predominance of necrosis centrally were retained as the control tumours grew in size from T to T + 3 days.

FIG. 2.—Mean relative proportions of the various tumour components shown in the key of Fig. 1 of the outer, middle and inner zones of control and CP treated tumours 0·5 days after T size. The number of tumours analysed to produce each diagram was at least 3.

Twelve hours after CP the number of labelled cells increased in each zone and the amount of necrosis had decreased (Fig. 2). Necrosis was still predominant in the inner zone whilst the greatest number of labelled cells was in the outer zone. One and 2 days after CP this general pattern of central necrosis and peripheral proliferation was maintained. In contrast to control tumours 0·5 hours after T size, Fig. 2 shows that at this time after CP almost half of the tumour nuclei in the inner zone were labelled.

The distribution of the tumour components in the three zones 3 days after CP and that in the corresponding controls is shown in Fig. 3. The amount of necrosis was roughly the same in each zone of the CP treated tumours and this contrasts with the pattern of necrosis in the control tumours. In the CP treated tumours the majority of labelled cells and the largest area occupied by apparently viable tumour tissue occurred in the inner zone. In the outer zone of treated and control tumours an appreciable proportion of the tumour was made up of normal fibroblast-like cells.

The variable composition of the tumours, expressed as the percentage of the elements in the tumour sections, is shown in Table I.

The number of tumours examined was usually 3 and as a result of considerable variation between tumours many of the standard errors shown in Table I are large, and many of the differences between control and CP treated tumours shown in Fig. 1 are not statistically significant. The Student *t* test showed that after CP there was a significant decrease in the percentage of viable tumour from 0·5 to

FIG. 3.—Mean relative proportions of the various tumour components shown in the key of Fig. 1 of the outer, middle and inner zones of control and CP treated tumours 3 days after T size. The number of tumours analysed to produce each diagram was usually 3.

TABLE I.—*Mean and Standard Errors of the Percentage of Tumour Sections Occupied by Viable Tumour, Necrosis and Blood, as Determined by the Morphometric Analysis,* 0·5, 1, 2 *and* 3 *Days After T Size. The Number of Tumours Analysed in Each Group was* 3 *Except for Groups Marked* * *where* 4 *and* † *where* 2 *Tumours were Analysed*

| | Treatment | Days after T size 0·5 | 1 | 2 | 3 |
|---|---|---|---|---|---|
| % Viable tumour | Control | 67·6±5·4 | 49·2±13·9 | 52·9±5·8 | 35·1± 2·0† |
| | CP | 80·3±3·9* | 44·1± 3·3 | 39·6±6·7 | 42·9±12·6 |
| % Necrosis | Control | 16·9±3·9 | 39·6±12·8 | 44·7±5·9 | 54·2± 3·3† |
| | CP | 10·3±2·0* | 36·5± 3·4 | 59·4±7·1 | 51·4±14·1 |
| % Blood cells | Control | 16·2±2·5 | 11·0± 1·6 | 2·4±0·3 | 11·6± 3·5† |
| | CP | 10·9±3·5* | 19·3± 1·4 | 1·0±0·4 | 2·9± 0·9 |

1 day ($P < 0{\cdot}1\%$) and this was accompanied by a significant increase in the amount of necrosis ($P < 0{\cdot}1\%$). There was significantly more blood in CP treated tumours one day after T size than in the appropriate controls ($P < 5\%$).

The morphometric results were calculated to show the mitotic and $^3$H-TdR labelling indices and these are compared in Table II with those obtained previously on tumour smear preparations. There was often reasonable agreement between the indices determined from sections and smears although some of the standard errors were quite large. However, the salient features described in the previous investigation, an increase in the $^3$H-TdR labelling index 12 hours after CP accompanied by a decrease in the mitotic index and followed 2 days after CP, by a rise in the mitotic index when the labelling index had returned to normal, were confirmed in this analysis of tumour sections.

### DISCUSSION

This morphometric analysis confirms our previous study on smear preparations made from tumour cell suspensions, on the changes in the proliferative pattern of the $RIB_5$ tumour after a single dose of CP (100 mg/kg body weight) (Peel and Cowen, 1972). It also quantifies the contribution of necrotic tissue and blood to the tumour mass.

The theory and value of morphometric analysis have been discussed by Chalkley (1943), Weibel, Kistler and Scherle (1966) and Schroeder and Münzel-Pedrazzoli (1970) and all agree that adequate sampling of the point observations will produce a representative analysis. We found that

TABLE II.—*The* $^3$*H-TdR and Mitotic Indices of Control and CP Treated Tumours* 0·5, 1, 2 *and* 3 *Days After T Size as Determined on Smears of Tumour Cell Suspensions and in a Morphometric Analysis of Tumour Sections. The Number of Tumours Analysed in Each Group was* 3 *Except for the Group Marked* * *where* 4 *and* † *where* 2 *Tumours were Analysed*

| | | T+0·5 Control | T+0·5 CP* | T+1 Control | T+1 CP | T+2 Control | T+2 CP | T+3 Control† | T+3 CP |
|---|---|---|---|---|---|---|---|---|---|
| $^3$H-TdR labelling index | Smear | 20·2 ±1·9 | 35·9 ±4·8 | 21·6 ±2·9 | 15·8 ±4·9 | 20·0 ±1·9 | 15·2 ±2·8 | 23·3 ±1·6 | 21·4 ±2·9 |
| | Section | 25·9 ±3·4 | 50·8 ±6·2 | 23·8 ±7·5 | 20·0 ±5·4 | 27·8 ±2·6 | 29·5 ±5·1 | 21·7 ±3·4 | 25·5 ±1·9 |
| Mitotic index | Smear | 1·7 ±0·2 | 0·5 ±0·2 | 1·0 ±0·1 | 0·5 ±0·2 | 1·4 ±0·1 | 4·0 ±0·7 | 1·9 ±0·2 | 3·7 ±0·7 |
| | Section | 2·0 ±0·2 | 0·7 ±0·2 | 2·5 ±0·5 | 0·7 ±0·2 | 2·4 ±0·8 | 3·6 ±0·3 | 1·0 ±0·2 | 2·3 ±0·6 |

about 1000 observations, in each of 3 zones of the tumour, taken from scans across at least 3 diameters gave results which were representative of the relative amounts of the various tumour components in each zone of that tumour section.

In control tumours (Fig. 1a) the amount of necrosis was shown to increase as the tumour grew and this was accompanied by a decrease in the amount of apparently viable tumour tissue. Thomlinson (1960), investigating the use of oxygen in radiotherapy and using the $RIB_5$ tumour as a model, cited John's hypothesis (Churchill-Davidson, Sanger and Thomlinson, 1957) which suggests that as a tumour grows the increase in necrosis is a result of progressive venous obstruction. Such obstruction would be accompanied by stasis in capillaries and eventual death of erythrocytes. In our analysis dead erythrocytes were scored in the necrotic element and this may be the reason for the decrease in the number of erythrocytes in control tumours in the 2 days following T size. The increase in the number of erythrocytes on the third day after T size may be because the balance between proliferation of new capillaries and the obstruction of old ones has altered. The relationship between tumour vasculature and tumour cell proliferation has been emphasized by qualitative and quantitative studies of their anatomy, function and necrosis (Rubin and Casarett, 1966; Tannock and Steel, 1969) and by Song and Levitt (1971) in a study of the relationship of tumour mass to functional intravascular volume and extravasation of plasma protein.

In treated tumours (Fig. 1b), the rise of the tumour cell $^3$H-TdR labelling index 12 hours after treatment with CP confirmed our previous results but it was accompanied by a fall in the proportion of necrotic tissue. The simplest explanation would be that the tumour cells increased in volume and were constrained by the fibrous capsule around the tumour and so compressed the necrotic elements. However, the explanation may be more complex because circulation through the tumour is probably changed by the CP and the rate of clearance of interstitial fluid and necrotic elements may be altered.

The rise in $^3$H-TdR labelling index 12 hours after CP was short lived. Between 12 and 48 hours after CP there was an increase in the amount of necrotic tissue, cell death was predominant but at 72 hours tumour cell proliferation was re-established.

The tumours were studied with respect to the changes in the inner, intermediate and outer zones. The controls showed the maximum number of labelled tumour cells in the outer zone and maximum necrosis in the centre of the tumour. The small proportion of tumour nuclei labelled in the inner zone contrasts with the large proportion labelled in the outer zone and shows the variability in labelling index from one region to another. Variation in proliferative activity at various sites within a tumour has been described for several tumours, *e.g.* Frindel *et al.* (1967), Hermens and Barendsen (1967, 1969) and Tannock (1968), and it is suggested that the major factor in the variation in proliferative activity is an alteration in the growth fraction.

Twelve hours after CP an increased proportion of tumour nuclei in all zones was made up of labelled nuclei. Between one and 2 days after CP the general pattern of central necrosis and peripheral proliferation remained unchanged. On the third day after CP it appeared that the amount of necrosis was approximately the same in all zones but tumour cells labelled with $^3$H-TdR were plentiful in the inner zone. This phenomenon of increased proliferative activity in the centre of the tumour may be similar to the altered pattern of proliferation described by Hermens and Barendsen (1969) in a rat rhabdomyosarcoma 4 days after x-irradiation.

If this central proliferation 3 days after CP can be confirmed in other tumour models it might suggest that an increase in cell killing could be achieved if the tumour was irradiated at this time after CP before the normal distribution of oxygenated and anoxic zones was re-established.

These two studies of the response of $RIB_5$ to cyclophosphamide have shown that the proliferative state of the tumour alters considerably during the 72 hours after giving the drug. However, knowledge of the tumour as an integrated unit of tumour tissue, necrotic and blood elements is obtained only from a detailed study of tumour sections. It is apparent that the part played by tumour vasculature in tumour growth and regression has yet to be fully understood and the response of a tumour and its blood vessels to therapy is of paramount importance in influencing the subsequent events of tumour growth and response to further treatment.

We thank Professor E. H. Cooper for his helpful discussions and Carol Nutman for her excellent technical assistance.

This work was supported by The Yorkshire Council of The Cancer Research Campaign.

### REFERENCES

Chalkley, H. W. (1943) Method for the Quantitative Morphologic Analysis of Tissues. *J. natn. Cancer Inst.*, **4**, 47.

Churchill-Davidson, I., Sanger, C. & Thomlinson, R. H. (1957) Oxygenation in Radiotherapy II. Clinical Application. *Br. J. Radiol.*, **30**, 406.

Frindel, E., Malaise, E. P., Alpen, E. & Tubiana, M. (1967) Kinetics of Cell Proliferation of an Experimental Tumour. *Cancer Res.*, **27**, 1122.

Hermens, A. F. & Barendsen, G. W. (1967) Cellular Proliferation Patterns in an Experimental Rhabdomyosarcoma in the Rat. *Eur. J. Cancer*, **3**, 361.

Hermens, A. F. & Barendsen, G. W. (1969) Changes of Cell Proliferation Characteristics in a Rat Rhabdomyosarcoma before and after X-Irradiation. *Eur. J. Cancer*, **5**, 173.

Peel, S. & Cowen, D. M. (1972) The Effect of Cyclophosphamide on the Growth and Cellular Kinetics of a Transplantable Rat Fibrosarcoma. *Br. J. Cancer*, **26**, 304.

Rubin, P. & Casarett, G. (1966) Microcirculation of Tumours. Part 1: Anatomy, Function, and Necrosis. *Clin. Radiol.*, **17**, 220.

Schroeder, H. E. & Münzel-Pedrazzoli, S. (1970) Application of Stereologic Methods to Stratified Gingival Epithelia. *J. Microsc.*, **92**, 179.

Simpson-Herren, L., Blow, J. G. & Brown, P. H. (1968) The Mitotic Cycle of Sarcoma 180. *Cancer Res.*, **28**, 724.

Song, C. W. & Levitt, S. H. (1971) Quantitative Study of Vascularity in Walker Carcinoma 256. *Cancer Res.*, **31**, 587.

Tannock, I. F. (1968) The Relationship between Cell Proliferation and the Vascular System in a Transplantable Mouse Mammary Tumour. *Br. J. Cancer*, **22**, 258.

Tannock, I. F. & Steel, G. G. (1969) Quantitative Techniques for Study of the Anatomy and Function of Small Blood Vessels in Tumours. *J. natn. Cancer Inst.*, **42**, 771.

Thomlinson, R. H. (1960) An Experimental Method for Comparing Treatments of Intact Malignant Tumours in Animals and its Application to the use of Oxygen in Radiotherapy. *Br. J. Cancer*, **14**, 555.

Weibel, E. R., Kistler, G. S. & Scherle, W. F. (1966) Practical Stereological Methods for Morphometric Cytology. *J. Cell Biol.*, **30**, 23.